# Improving Sexual Health Education Programs for Adolescent Students through Game-Based Learning and Gamification

**DOI:** 10.3390/ijerph15092027

**Published:** 2018-09-17

**Authors:** Hussein Haruna, Xiao Hu, Samuel Kai Wah Chu, Robin R. Mellecker, Goodluck Gabriel, Patrick Siril Ndekao

**Affiliations:** 1 Faculty of Education, University of Hong Kong, Pokfulam Road, Hong Kong, China; xiaoxhu@hku.hk (X.H.); samchu@hku.hk (S.K.W.C.); robmel@hku.hk (R.R.M.); 2 Animation Creation Department, Innovative Development Tanzania, 63 Galu Street, Ada Estate, Dar Es Salaam, Tanzania; goodluckgabriel201@gmail.com; 3 Directorate of Library Services, Institute of Finance Management, 5 Shaaban Robert Street 11101, Dar Es Salaam, Tanzania; cyrilbab@gmail.com

**Keywords:** sexual health education, sexual well-being, adolescent students, game-based learning, gamification, MAKE framework, randomized controlled trial, reduction unhealthy sexual behaviour, prevention STIs and HIV/AIDS, digital health technologies

## Abstract

An effective innovative pedagogy for sexual health education is required to meet the demands of technology savvy digital natives. This study investigates the extent to which game-based learning (GBL) and gamification could improve the sexual health education of adolescent students. We conducted a randomized control trial of GBL and gamification experimental conditions. We made a comparison with traditional teaching as a control condition in order to establish differences between the three teaching conditions. The sexual health education topics were delivered in a masked fashion, 40-min a week for five weeks. A mixed-method research approach was uses to assess and analyze the results for 120 students from a secondary school in Dar Es Salaam, Tanzania. Students were divided into groups of 40 for each of the three teaching methods: GBL, gamification, and the control group (the traditional teaching method). The average post-test scores for GBL (Mean = 79.94, SD = 11.169) and gamification (Mean = 79.23, SD = 9.186) were significantly higher than the control group Mean = 51.93, SD = 18.705 (*F* (2, 117) = 54.75, *p* = 0.001). Overall, statistically significant differences (*p* ≤ 0.05) were found for the constructs of Motivation, Attitude, Knowledge, and Engagement (MAKE). This study suggests that the two innovative teaching approaches can be used to improve the sexual health education of adolescent students. The methods can potentially contribute socially, particularly in improving sexual health behaviour and adolescents’ knowledge in regions plagued by years of sexual health problems, including HIV/AIDS.

## 1. Introduction

The increase in unhealthy sexual behaviors among adolescent students has sparked alarm and has become an area of interest for global public health researchers aiming to find innovative approaches to promote better healthy sexual outcomes. During adolescence, young people undergo physical, mental, and emotional changes brought about by increased hormonal function. These biological changes increase interest in sexual behaviour and leave adolescents vulnerable [1]. Many young people are susceptible to risky sexual behaviors such as engaging in underage sexual intercourse, polyamory, participating in unprotected sex, and exposing themselves to potential sexual assault environments [2]. Substance use, which contributes to unhealthy sexual behaviour, has been found to be common and increasing among adolescents [3]. These behaviors adversely affect their future health outcomes, increasing their chances of contracting sexually transmitted infections (STIs) including HIV/AIDS; dropping out of school due to unplanned pregnancies [1]; and contracting human papillomavirus (HPV) [4,5], which contributes to contracting cervical cancer [6,7,8].

Sexual health education has great potential for providing the knowledge and skills necessary for adolescents to make safe choices related to sex [9]. It can reduce misinformation and increase critical thinking, communication, and self-confidence. These will lead to young people making smarter choices regarding their sexual relationships. Comprehensive sexual health education and implementation strategies have been recognized and developed [10]. The knowledge obtained helps adolescents to reduce their chances of engaging in risky sexual behavior. These sexual health programs are developed to accommodate different populations. For example, while school-based sexual health education targets adolescents in formal education systems and is incorporated in school curricula [11], there is also a program designed to reach adolescents who are disconnected from the school system [12]. Both approaches have been effective in promoting healthy sexual behaviour of adolescents, allowing them safe passage to an adulthood where they can achieve their full potentials and realize their goals.

Despite the various sexual health educational initiatives, the delivery and implementation of sexual health education is still controversial in resource-limited settings. Irresponsible adolescent sexual health behaviour and the resultant adverse health effects are on the rise in developing countries [9,13,14]. The inadequate comprehensive adolescents’ sexual well-being knowledge and skills make them more likely to engage in unhealthy sexual behavior [15]. In Sub-Saharan Africa (SSA), most adolescents have little knowledge about sexual and reproductive health [14]. For example, in 2015, the report on HIV AIDS in SSA showed that adolescents aged 15–19 years accounted for 37% of new infections [16]. These alarming statistics have resulted in appeals for public health interventions to address the increasing level of STIs (including HIV/AIDS) and other risk sexual behaviors, and to improve and implement sexual health education initiatives [17].

The inadequate implementation of sexual health education in school is due to various factors, including social and cultural attitudes that do not allow open discussion and teachers’ resistance to teaching sexual health matters [14]. Furthermore, the existing, widely employed traditional teaching methods do not support the effective delivery of sexual health education [13,18]. Adequately accessible sexual health education could help to protect adolescents. In this study, interventions are designed, tested, and evaluated for developing countries, where sexual behaviour problems of adolescents are increasing, resulting in teenage pregnancies and extremely serious chronic diseases.

### 1.1. Serious Games as a New Pedagogy

The application of information technology (IT) in education has been found to be an innovation enabling effective behaviour change and knowledge acquisition. Thus, the advancement of IT in health could be of benefit to sexual health education, particularly in the development of interventions that involve the use of “serious games” [19]. In sexual health education, serious games have been acknowledged to be appealing to adolescents as they are attractive, confidential, and convenient, and can avoid the embarrassment or boredom of discussing the issues with teachers or health promotion educators [20,21]. Scholars [22,23] have identified eight characteristics to be considered when designing effective sexual health education games: (1) individual tailoring, (2) goal setting, (3) narrative or storyline, (4) audio-visual effects, (5) interactivity, (6) challenges at each level, (7) rewards, and (8) immediate feedback.

Adolescents are currently exposed to the digital games environment, and thus the majority of them are familiar with and able to play games for enjoyment and fun [24,25]. Thus, for sexual health matters, games can be an easier and more motivating method of educating [21,26] than traditional teaching methods [13]. They offer opportunities for promoting safer sexual behaviour through a personalized learning environment, which can deliver potential messages [27] through relevant storylines, role-playing, and avatars. Apart from enjoyment while learning, the use of games for sexual health education has been reported to reinforce adolescents’ healthy sexual decision-making and to refrain from engaging in risky sexual behaviour [28]. This teaching approach has previously been shown to transfer sexual health knowledge and transform problem-solving skills into making informed decisions regarding sexual health matters [13].

Sexual health knowledge acquisition through games can also enhance cognitive development, promote and raise awareness, and encourage behavioural change [29]. Learning through games is confidential by nature, hence encouraging adolescents to discuss sexual health matters freely [30]. Therefore, it is applicable even in cultural contexts such as Africa, where talking about sex matters in public is taboo. Sexual health education through games enables adolescents to easily absorb, transfer, and retain the intended information [31]. The learner is engaged to act in various challenging learning activities (thinking tasks, quizzes, competitions) that foster cognitive functioning and development skills including critical thinking, decision-making, remembering, evaluating, reasoning, and problem-solving.

### 1.2. Game-Based Learning

Game-based learning is the application of any game-based approach designed with the main purpose of delivering learning rather than entertainment [32]. Empirical studies [19,33] have shown the potential of GBL for adolescent sexual health education. Digital game technology has great potential for educating today’s digitally-oriented adolescents about health. In particular, digital health games can make the promotion of sexual well-being of adolescents more effective. There has been a rapidly increasing use of digital games to promote adolescent sexual well-being [21,31,34]. Digital health games increase engagement and make the learning environment more interactive. They also offer practical skills through hands-on learning activities which are translatable to the real world [35]. Their repetitive nature is ideal for learning. The games provide immediate feedback, which is highly beneficial to the learner [33]. Interest is emerging in developing digital health games for sexual health education and evaluating their effects [36]. Digital health game interventions have been shown to affect the sexual behavior of adolescents. Some digital game interventions have focused on the general promotion of sexual health [21,37], some have focused on HIV/AIDS prevention [35], some have focused on preventing the spread of Chlamydia and other sexually transmitted infections (STIs) [31], some have focused on encouraging human papillomavirus (HPV) vaccinations [38], and others have aimed at eliminating coercion and pressure in adolescent relationships [39]. Although GBL has shown a great potential in sexual health education, little is publicly known about its application and its effectiveness in the study context (developing countries, which have limited resources). This study aims to fill that gap.

### 1.3. Gamification

GBL and gamification both involve game mechanics. Gamification is the application of digital game mechanics in a non-gaming context for the purpose of engaging learners, motivating activities, enhancing learning, and solving problems [40]. For example, the Moodle learning platform is a non-gaming context, and game mechanics are readily available. The common game mechanics are points systems, leaderboard positions, badges, trophies, achievements, competitions, and levels [41]. In learning, gamification could be employed for: (1) narratives that amend the setting for a certain task; (2) the design of social competition; and (3) the provision of incentives, such as badges and rewards, that encourage behavioural change [42]. Gamification has potential in the health education of adolescents [43]. It could be effective in encouraging student participation, which is essential for influencing the acquisition of the knowledge and skills needed for attitude and behavioural change. It offers opportunities for students to be engaged, motivated, and enjoying themselves while learning [44,45]. Gamification is an innovative learning approach that could make sexual health education more effective. For example, study [46] reports game mechanics as effective in minimizing sexual and relationship violence among adolescents. Another empirical study [47] demonstrates that the elements of gaming have significant effects on STIs and on an HIV campaign that reached a large population of young adults. Although gamification has shown great potential in sexual health education, little is publicly known about its application and effectiveness in the study context (limited-resource countries). This study aims to fill the gap.

### 1.4. Theoretical Framework

**Figure 1:**
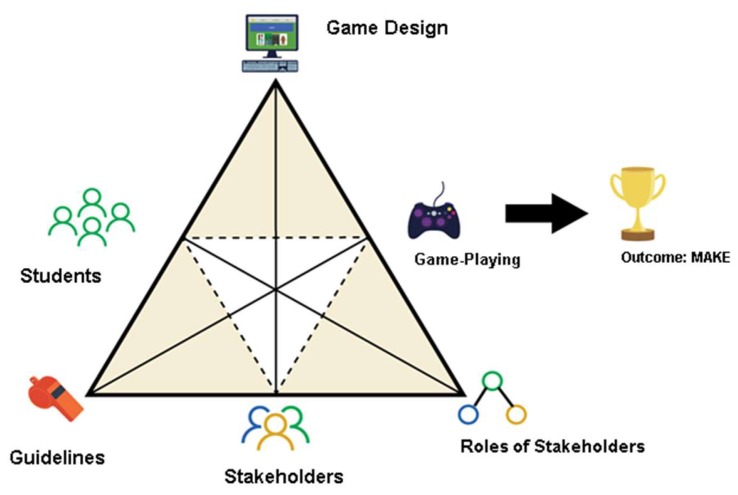
The expanded game activity system framework suggested by Engeström [50].

Activity Theory (AT) has been previously used as a framework for the research and design of GBL and gamification instructional interventions and to inform knowledge construction as a social practice [48]. The AT framework consists of instruments, subjects, objects and outcomes, rules, community, and division of labour [49]. In this study, secondary school adolescents (the subjects) use GBL and gamification teaching methods (the instruments) to access sexual health learning content in interactive manner in a way that inculcates habits that curb imprudent sexual behaviour (the outcome). Figure 1 presents the adapted game activity system as the theoretical framework used in this study [50].

This study uses the comprehensive and flexible sociocultural learning theory, Activity Theory (AT). We developed game mechanics known to be successful educational tools (storylines, scenes, characters, settings, environments, points, badges, leaderboards, and choices) [51] for the GBL and gamification educational platforms. We engaged various stakeholders (the community) to participate in different roles (division of labour) to design and develop the GBL and gamification methods that help students acquire knowledge (the object), which is crucial toward achieving a better sexual health outcome (the goal). It is important to note that we placed much emphasis on community engagement when designing, developing, testing, and refining the intervention. While learning, students adhered to the norms and guidelines (rules) [52] that govern game mechanics such as gaining points, badges, leaderboards, and gameplay.

### 1.5. Current Study

The delivery of sexual health education to students in SSA countries is characterized by traditional teaching methods, which are limited to lecturing with little or no feedback from the teacher and no room for discussion. The assumption is that all students can learn at the same pace and are active listeners [13]. This traditional method could be a key contributing factor to ineffective sexual health education programs for adolescents in SSA secondary schools [53]. Digital games have been confirmed to be effective in transferring sexual health education and behavioral knowledge among adolescent students [21]. However, learning content and game elements that adhere to the social and cultural background of the society should be carefully selected, and focus on existing cultural and ethnic norms. Unlike other teaching methods, game-based teaching can be designed with specific socio-cultural content when delivered in countries where sex education discussions in public are taboo [30].

Given the reported ineffectiveness of national sexual health education [18,54] and the advances and success of GBL and gamification pedagogies [13], we aimed to investigate the extent to which GBL and gamification could improve the teaching of sexual health education to secondary school adolescent students. To determine the effectiveness of these methods when compared with the traditional teaching method, we aimed to determine learning performance through sexual health literacy tests and assess students’ Motivation, Attitudes, Knowledge gain, and Engagement using the MAKE model [55]. Based on the above aims, our hypotheses for this study were twofold: (1) We hypothesized that sexual health education would be more efficient if taught using GBL and gamification than traditional teaching methods; and (2) we hypothesized that GBL and gamification teaching methods would be more effective with regard to students’ Motivation, Attitudes, Knowledge gain, and Engagement in sexual health education when compared with traditional sexual health education practices.

## 2. Materials and Methods

### 2.1. Research Design

We conducted a randomized controlled trial in one public school in the city of Dar es Salaam, Tanzania. Tanzania is an appropriate study setting as it is a signatory to various international and regional conventions that promote adolescent sexual and reproductive health. The research team developed and evaluated GBL and gamification interventions and compared their effectiveness with the existing traditional teaching method (Table 1). The details of the development of the digital health game are written about in another article. The Ministry of Health in collaboration with the Ministry of Education promotes sexual health education through the National School Health Program. Sexual health education is included in all National School curricula and is a compulsory subject for primary and secondary school students. Approval for ethics clearance for this research was obtained from the ethics panel of the Human Research Ethics Committee (HREC) of The University of Hong Kong with reference number EA1610018 and The National Institute for Medical Research (NIMR) in Tanzania.

### 2.2. Participants

This study focused on adolescent sexual health education for lower secondary schools, in particular, Form I students. A total of 120 lower secondary school students (11–15 years of age) were recruited to participate in this study. The students attended Form I education (equivalent to Grade 7 in the North American education system). Students were from three classes with equal educational status. Students in each class participated in one of three conditions, namely GBL, gamification, and traditional teaching. The treatments were randomly assigned for each class for the minimization of possible biases or judgements. Each condition was thus comprised of 40 students who were not exposed to the teaching method of the other classes or conditions. A masked fashion was used to eliminate the potential contamination of the conditions. Also, the training was conducted during school hours, hence each condition was in class for the session. Experimental conditions were given log-in codes for accessing learning content in the school computer lab. The content in the experimental condition was installed on the computer and was accessed offline. The learning content remained constant for all conditions and followed the curriculum used to teach adolescents about sexual and reproductive health, as stipulated by the school curricula and syllabi. The educational content included the following subjects focusing on adolescents: (1) personal hygiene and good manners; (2) sexual responsibility and decision-making; (3) dealing with peer pressure; (4) prevention of STDs, STIs, HIV, and AIDS; and (5) how to deal with harmful practices and sexual violence. Consent to involve students in the study was sought beforehand from relevant authorities, including school administrators and parents. No parents refused their child’s participation.

### 2.3. Teaching Methods (Procedure)

To improve the learning of all three of the conditions, we developed activities for teaching and learning using the participatory approach [21]. We invited individuals with expertise in sexual and reproductive health, sexual health education teachers, students, researchers, and computer and information scientists to participate in the development of teaching materials for sexual health education through GBL. The school curriculum schedule was followed in all three teaching conditions. The five-week sexual health education topics were delivered once a week for 40-min. In the traditional teaching method, teachers taught the lessons in the classroom, whilst GBL and gamification classes were conducted in a computer lab. Students in the experimental conditions accessed intervention contents on computer platforms offline. There was no possibility of accessing it outside of the school computer lab. Each participating student was given a code for accessing the learning contents.

Forty students were assigned to each condition. Each participant in the GBL or gamification conditions was assigned a computer to use during the lessons and was encouraged to learn independently. Students in these conditions were assigned accounts for accessing corresponding prospective digital software and Internet systems. The GBL and gamification groups were then given a 30-min orientation session before the first class to respectively learn about the game and about Moodle, a widely used learning management system [42,56,57,58]. Both the teacher and the first author of this article were present during these conditions to provide guidance when needed. All other conditions were kept constant for the three groups throughout the study.

#### 2.3.1. Traditional Teaching

The traditional teaching approach widely used in secondary school education [13,18,59] was used in this study. The Form I teacher followed the school schedule for sexual education instruction and conducted traditional sexual health education in the classroom according to the school curricula. Several activities took place during classroom teaching, such as class discussions, group buzzing, and group work, in addition to individual assignments. Sexual health education teaching content was also made available to the students in study areas. The teacher assessed students’ activities through discussions and marked their individual assignments. These traditional classroom teaching methods had no supporting information technology tools.

#### 2.3.2. Game-Based Learning

**Figure 2:**
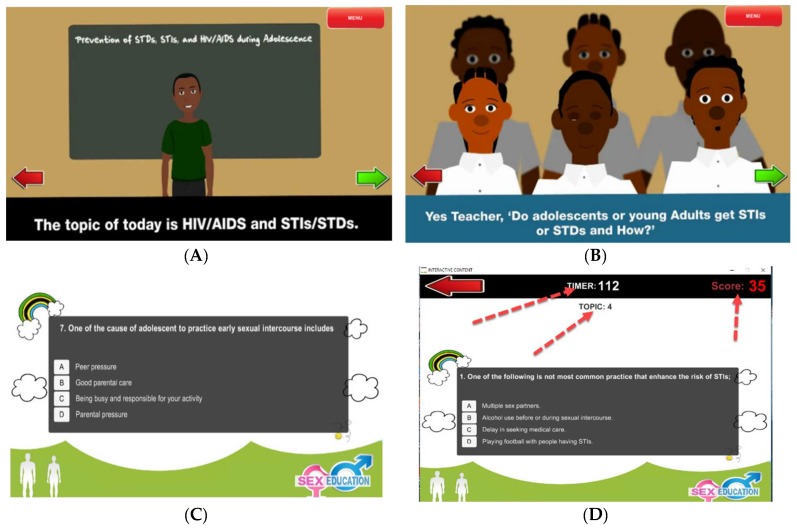
GBL condition game structure. (**A**) Scenario: teacher presenting the topic; (**B**) Scenario: students attending the class; (**C**) Questions; (**D**) Activity time, topic, and scores.

The research team developed the GBL in collaboration with end users and experts from various fields (Figure 2). As informed by design-based research [60], before the development of the interventions, we conducted a situational analysis of the practical problems, which involved the collaboration of researchers, reproductive health specialists, computer and information scientists, community members, teachers, and students as users of the interventions. The game was developed based on the above collaborations [21,61,62] and contributions by secondary school students (junior and senior) from another school. We asked students to reflect upon and share their stories, experiences, and views on what they would like this game to be. Students also proposed the game characters suitable for the study context and messaging. Digital stories were woven into the learning content and embedded in an interactive manner. The scenario game was developed using UNITY 5, manufactured by Unity Technologies, San Francisco, California, United States [63].

Students played the game individually, performing various activities such as attempting quizzes and completing exercises related to sexual education. The GBL students were asked to view the game story and later attempt series of questions for each topic. To move to the next topic, students were required to score at least 6 out of 10 points. If student failed to achieve 6 points, the game was restarted from the beginning.

#### 2.3.3. Gamification

**Figure 3:**
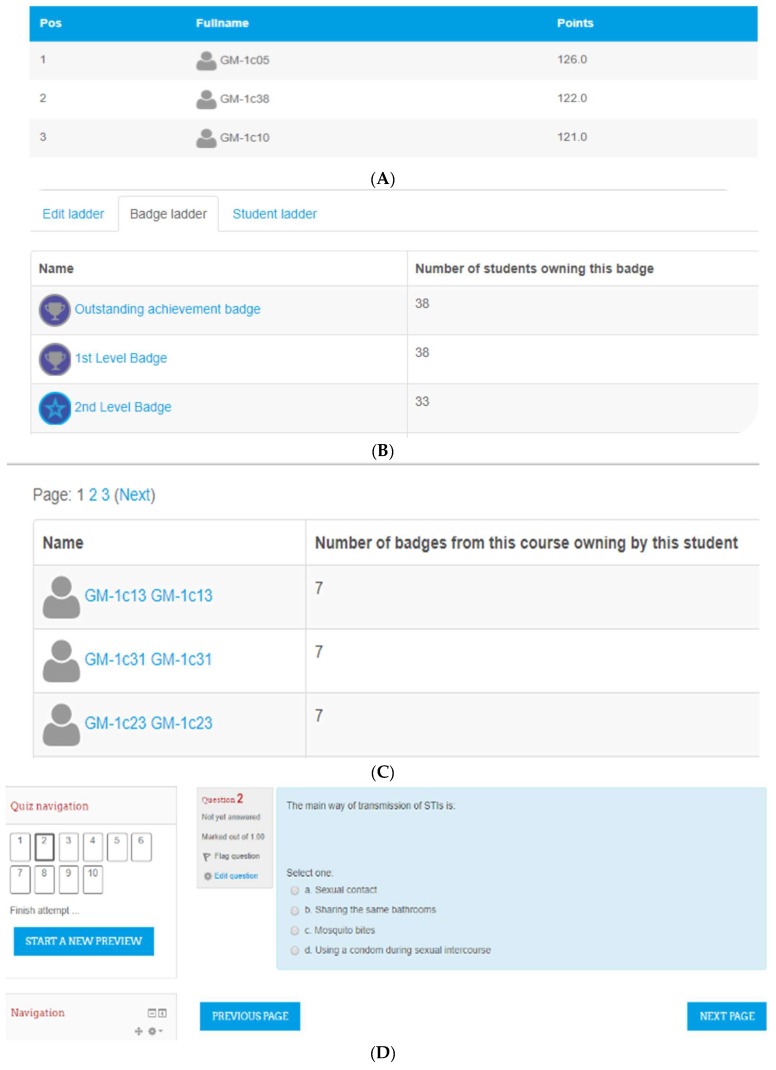
Game mechanics used in the gamification condition. (**A**) Leaderboards; (**B**) Badges; (**C**) Badges counter; (**D**) Quiz.

In the gamification condition, sexual health education was gamified through game mechanics, including badges, leaderboards, and a point system [46]. Gamification plugins are readily available on Moodle [56], which enabled this study to use the Moodle platform to implement game mechanics with learning content (Figure 3). Students were provided with the sexual health education lessons in a quiz format. Each topic was represented by one quiz containing 10 relevant questions. Students were asked to answer the questions and were awarded points for correct answers and lost points for incorrect answers. Badges were awarded to students with the highest scores. This award system was constructed to create competition and a desire to continue to learn more content [41,42].

### 2.4. Measures

#### 2.4.1. Adolescent Sexual Health Literacy Tests

In collaboration with school teachers and a reproductive health specialist, a set of sexual health knowledge questions covering the five topics of sexual and reproductive health, known as the Adolescent Sexual Health Literacy Test (ASHLT), was developed. For each topic, there were 10 questions and each question were assigned 2 marks, resulting in a possible score of 100. To assess prior knowledge and experience about sexual and reproductive health, a pre-test knowledge assessment was administered before the beginning of the class (one week before the intervention). The knowledge assessment consisted of three sections: Section A (multiple choice); Section B (true/false); and Section C (short answer). Students were given up to 45-min to complete the questions. To evaluate the acquisition of knowledge from the teaching methods, all students were given a post-test within one week after the intervention that contained the same questions attempted during the pre-test. The questions were in the same order as those presented in the pre-test.

#### 2.4.2. Evaluation of Teaching Approaches

**Figure 4:**
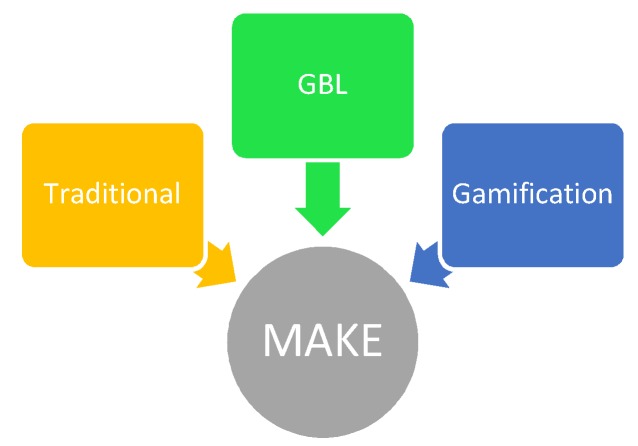
Evaluation of three teaching methods using the MAKE framework.

The unified MAKE framework has been used separately [21,29,64,65,66,67,68,69,70] to evaluate the effectiveness of designed instructional interventions in sexual health education. This study unified four constructs to develop a comprehensive MAKE framework used to evaluate the effectiveness of the three teaching methods [55] (see Figure 4). The assessment tools were adapted from previous studies [71,72,73] and modified to suit the current study objectives. The MAKE framework was comprised of 46 items, with 16 items in Motivation and 10 in each of the three remaining constructs (Attitude, Knowledge, and Engagement) (Appendix A). Each item was rated on a five-point Likert scale ranging from 1 (Strongly Disagree) to 5 (Strongly Agree). Details of the instrument development, reliability, and validation of the MAKE framework are provided in another article, which has been accepted for publication [55]. Within one week after the intervention, the students received a questionnaire about the efficacy of the three teaching methods based on the MAKE framework [55]. Students were given 15 min to complete the questionnaire.

#### 2.4.3. Focus Group Interview

Following the instructional evaluation, 21 students were invited to participate in focus group interviews (FGI) to collect feedback on experiences with the three teaching methods. Students were randomly selected from each condition to participate in one of three FGIs representing each condition. Each semi-structured FGI was comprised of six to eight students as recommended by Vaughn et al. [74]. The FGIs were significant as they provided opportunities for the students to express views and for the research team to obtain more information regarding the efficacy of each teaching method [75]. The intention was to explore and elicit the expression of student thoughts and perceptions on the three teaching conditions used in learning sexual health education in an open and unrestricted discussion within a comfortable environment [76]. The semi-structured interview protocol was informed by the MAKE framework [55]. Students were asked about their perception of the efficacy of the teaching methods and how they felt the methods influenced their learning of sexual health education. The main questions asked were how and why the teaching methods could: Motivate learning, promote a change of Attitude, increase Knowledge gain, and Engage students. The interview data were audio-recorded and transcribed verbatim using special codes (pseudonyms) for subsequent analysis. To control over-dominance by talkative students, each group member was given roughly an equal time over the one-hour session to comment on his/her views. Member validation was used to make critical comments on the adequacy of the results.

### 2.5. Data Analysis 

IBM SPSS Statistics for Windows, Version 24.0, Armonk, NY, USA: IBM Corp. was used to analyze quantitative data collected from ASHLT and the MAKE framework. A paired *t*-test was used to compare pre-test and post-test scores, to discover differences in test scores before and after the intervention. A one-way ANOVA was conducted to analyze numerical data obtained from pre-tests and post-tests and to compare means across groups. The comparison of pre-test scores was conducted to establish possible differences between the three groups prior to the intervention and rule out participant bias. Descriptive statistics were used to generate mean, median, and standard deviation variables for each of the four MAKE evaluation constructs. The Kruskal-Wallis test was used to analyze the ordinal data obtained from surveys and make comparisons across the three teaching conditions. Interview transcripts were selected to supplement and corroborate the quantitative results. The themes were developed based on the MAKE framework. See Appendix B for details about the FGIs protocol.

## 3. Results

### 3.1. Participant Baseline Characteristics

A total of 120 students were recruited and participated in this study. Details of their demographic information, social economic status (SES), and the rate of access and use of digital technologies are presented in Table 2. 

### 3.2. ASHLT Measures

**Figure 5:**
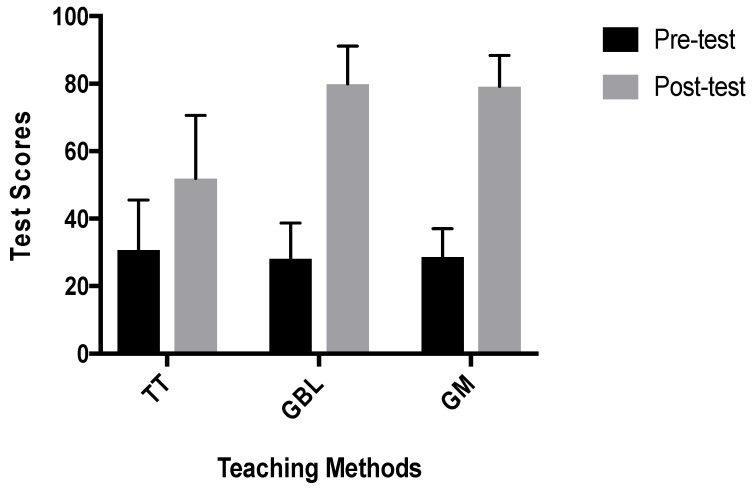
Mean (SD) test scores for the three teaching groups. Notes: The mean difference indicates significance at the *p* < 0.05 level (TT: Traditional Teaching method; GBL: Game-Based Learning; and GM: Gamification).

The paired sample *t*-test conducted on the ASHLT indicated a statistically significant increase in ASHLT average scores from pre-test (Mean = 29.26, SD = 8.689) to post-test (Mean = 70.36, SD = 18.201) (t (119) = −23.787, *p* = 0.001 (2-tailed)). The mean increase in the ASHLT scores was 41.10. A one-way ANOVA was used to compare participant pre-test and post-test scores on their sexual health knowledge between the three teaching methods. As shown in Figure 1, pre-test scores were similar, indicating that participants were equally distributed in all three teaching methods (F (2, 117) = 0.556, *p* = 0.575). The average post-test scores reported from the experimental conditions increased more than those of the control conditions (Figure 5). A statistically significant difference was found between the post-test scores under the three conditions (*F* (2, 117) = 54.75, *p* = 0.001).

Post-hoc tests were used as a follow-up analysis to determine the differences in the three pairs of teaching conditions. Significant differences were found between traditional and GBL (*p* = 0.001) and between the traditional and gamification pairs (*p* = 0.001). There was no effectual difference between the GBL and gamification pairs (*p* = 0.970). The results suggest that the instructional approach used in the GBL and gamification experimental conditions had a greater positive impact on students’ sexual health knowledge gains and understanding than students in the traditional conditions.

### 3.3. Evaluation of Teaching Methods with MAKE

#### 3.3.1. Motivation

The attention, relevance, confidence, and satisfaction (ARCS) components of motivation [77] were used in this study to assess the effectiveness of the designed instructional interventions in increasing and sustaining motivation during learning. As summarized by Huang et al. [77] each ARCS component evaluates different categories of motivation as follows: Attention: perceptual arousal, inquiry arousal, and validity; Relevance: goal orientation and motive matching; Confidence; learning requirements, success opportunities, and personal responsibility; and Satisfaction: intrinsic reinforcement, extrinsic rewards, and equity. The results from the ARCS component analysis are presented in Table 3. Students in the GBL and gamification conditions rated higher on average than students in the traditional teaching condition for all motivational constructs. Follow-up pairwise comparison tests were conducted for the three pairs of teaching methods. Pairwise comparisons revealed differences between the traditional and gamification condition pairs (*p* = 0.001). This effect remained similar for the traditional teaching and GBL pairs (*p* = 0.001). There was no evidence of differences between the gamification and GBL conditions pairs (*p* = 1.000). The comparisons between the three pairs of teaching conditions for relevance, confidence, and satisfaction components were similar.

According to these results, students attending sexual health education through GBL and gamification were more motivated than those in the traditional teaching approach. During the focus group discussion (FGI), students supported the assumption that both GBL and gamification motivated them during learning. A student (coded GBL-8) in the GBL condition stated that “*learning was more friendly and fun than other courses that used traditional teaching approach.*” Another participant (GM-19) in the gamification condition reported that “*learning was easier than other courses,*” while another in the same condition (GM-5) claimed that “*there was more freedom of learning.*” In contrast, negative opinions were revealed by the students in the traditional teaching method, such as “*some teachers come in the class with bamboo or wooden sticks for beating us*” (TT-13). “*We are uncomfortable as we fear for being beaten*” (TT-10), and “*I feel like we are not free to learn*” (TT-1).

#### 3.3.2. Attitude

Two attitude components were evaluated: an affective attitude was measured by students’ emotions or feelings toward teaching methods; and a cognitive attitude was measured by their attitudes toward the intellectual content, such as the facts and information taught by the three teaching methods [78,79,80]. The overall average of students was higher for self-ratings of affective attitude toward gamification than for traditional teaching students (Table 4) and was similar for the cognitive attitude. A non-parametric Kruskal-Wallis test indicated that differences achieved significance between all attitude components and teaching methods (*p* = 0.001). Following the Kruskal-Wallis test, pairwise comparison tests were conducted to determine the difference between the three pairs of teaching methods in relation to attitude components. The differences between traditional and GBL pairs (*p* = 0.001) and between traditional and gamification pairs (*p* = 0.001) are shown in Table 3. The GBL and gamification pairs did not significantly vary (*p* = 0.351) and the results from the cognitive attitude component were consistent.

The above quantitative analyses are further supported by qualitative data obtained from focus group interviews. In the FGI, GBL and gamification methods elicited greater feelings of positive attitude. The GBL students stated, “*I was interested in the instructional approach*” GBL-20) and “*My participation to the course was very high due to this teaching approach*” (GBL-16)*.* The gamification students voiced their positive thoughts accordingly: “*This teaching method is worthwhile for teaching this subject*” (GM-19). However, students in the traditional teaching method class appeared to have negative experiences: “*I feel shy, embarrassed, and awkward to ask questions in sexual health matters in the class*” (TT-9) and “*When teacher comes in the class to teach sexual and reproductive health some students start to laugh and giggle*” (TT-22).

#### 3.3.3. Knowledge

In addition to the knowledge outcome obtained from the ASHLT scores, this study also evaluated students’ perception regarding the efficacy of the teaching methods and the components associated with knowledge gain. Table 5 summarizes the descriptive statistics for the knowledge gain components and teaching methods. Students in the gamification or GBL condition rated higher for knowledge components than those in the traditional teaching condition. On the importance of knowledge, students scored higher under gamification and GBL methods compared to traditional teaching methods. The Kruskal-Wallis test indicated statistically significant differences among the three teaching methods for all knowledge components (importance of knowledge, effectiveness of knowledge, and application of knowledge) (*p* = 0.001). Further, pairwise tests indicated significant differences between the traditional and GBL or gamification methods for all three components (*p* = 0.000). Differences between the GBL and gamification pairs were insignificant (*p* = 1.000).

Overall, students in the GBL and gamification groups appeared more satisfied with their teaching approach in imparting sexual health knowledge than those in the traditional teaching method group. However, though all students agreed that the three teaching methods can be used to teach sexual and reproductive health issues, their views of their effectiveness differ, as a participant in the gamification condition reported that “*It is easy to understand and catch up learning*” (GM-2). In the GBL condition, a participant stated that “*I enhanced my understanding of sexual and reproductive health that can help me to curb irresponsible sexual behaviour*” (GBL-4). One participant suggested that the teaching method for enhancing knowledge acquisition in traditional teaching was ineffective, reporting that “*Sometimes the teaching and learning environment in the classroom is not conducive, therefore it is difficult to catch up on the intended knowledge*” (TT-40).

#### 3.3.4. Engagement

Questions related to engagement were used to evaluate the engagement aspect of teaching methods, including consistency, active participation, confidence, fun, excitement, individual attention, clarity of learning, meaningful work, rigorous thinking, and performance orientation [81,82]. Two engagement components emerged: emotional engagement, which evaluates students’ emotional reactions toward teaching methods; and cognitive engagement, which assessed students’ thinking investment in learning, including inspiration and self-regulation. Effective teaching methods that are engaging may affect students’ learning of sexual health subject matters. The descriptive statistics between engagement constructs and teaching methods are presented in Table 6. Students in the traditional teaching method reported that “*we are not free during learning as we feel shy and fear to ask questions in the class. As a result, the learning becomes unappealing and boring*” (TT-13) and “*The way we were taught this subject is too personal and teacher centered and but it is supposed to be more engaging, involving, and attractive*” (TT-18)*.* In contrast, students in the GBL teaching method explained, “*I enjoyed learning, I think even my fellow students felt the same way*” (GBL-21)*.* ”*I wanted to spend more time when the training ended*” (GBL-30)*.* In the gamification condition, students reported, “*I always am the last students to leave the computer lab and sometimes I was reluctant to shut off my computer*” (GM-5).

## 4. Discussion

### 4.1. ASHLT

The aim of this study is to investigate the extent to which GBL and gamification can improve the sexual health education of secondary students. Although 31.7% reported having no access to computers, 45% had no access to smart devices, and 35.8% had never having played digital games, these facts did not affect the experiments. Students were given an orientation on how to use the digital games for learning. Students in the experimental conditions provided opportunities for accessing the computer lab during their free time. Most of the students were fast learners and enjoyed the process. We could tell this even from evaluating the ASHLT results. Based on our knowledge-acquisition test, which evaluated knowledge acquisition before and after the interventions, students attained higher scores when the GBL and gamification teaching methods were used than when traditional teaching methods were used.

Our results concur with the results of sex education knowledge transfer in Hong Kong adolescents. Chu [21] used similar participatory approach methods to design a game, and in both studies, games were designed in the context of socio-cultural norms. This is important for knowledge acquisition as it contains specific aspects that can influence players to engage in learning activities while enjoying the learning process. Consequently, the information is easily absorbed, transferred, and retained [31]. Digital games are confidential in nature, and they may encourage learners to discuss sexual topics regarded as confidential in some cultural backgrounds [30]. Sexual health matters are not publicly discussed in the study context, and GBL- and gamification-based approaches allow them to learn and ask questions at their own pace and explore the subject of sexual health education in a private setting, without humiliation or asking questions that offend others.

Our positive outcome indicates that GBL and gamification teaching methods could effectively be used to teach sex education to secondary school students and with continued development, there is a possibility that these innovative methods may result in healthy behaviour changes. Game-style learning encourages students to acquire more knowledge about sexual health matters, and to potentially change risky sexual behaviors [83]. Games designed by researchers in the US to increase knowledge of Chlamydia for at risk youth were successful in transferring knowledge about STI’s and encouraging at risk youth to be tested for possible infections [31]). This study is in contrast to the current study in terms of the socio-cultural context (conducted in a developed country). However, the authors point out the inherent advantages of learning games (“relaxing and safe learning environment”), which were highlighted by the students in our focus group interviews.

### 4.2. MAKE Framework

In this study, key constructs of learning were investigated using the MAKE framework (motivation to learn, student attitudes change, knowledge attainment, and student engagement) and consequently shed light on possible mediating factors for the transfer of knowledge from GBL and gamification teaching methods.

#### 4.2.1. Motivation

Students reported greater levels of satisfaction for the GBL and gamification teaching methods than the students assigned to the traditional teaching method condition. One possible explanation for the higher levels of satisfaction of students in the GBL and gamification groups was the opportunity to interact and compete in attempting different tasks and the rewards offered through game mechanics [56]. The students in the GBL and gamification methods may have been supported by both intrinsic motives and extrinsic reward factors [72]. Competition, which is inherent in games, is a natural human trait and an intrinsic behaviour that drives the desire to accomplish a task with greater satisfaction and higher quality outcomes [84,85]. The game mechanics encouraged learners to compete in learning activities to achieve a set goal. Extrinsic motivations are external forces that drive behaviors in the pursuit of punishment or rewards [86]. The game mechanics (leaderboards, levels, and points) noted as extrinsic motivational factors may have played a role in driving students to be motivated in the learning process by gaining points, increasing levels, and reaching a rank on the leaderboards.

The qualitative data obtained from FGI provided further evidence that the students engaging in the GBL and gamification teaching methods were more motivated than those participating in the traditional teaching method. Students reported that GBL and gamification provided a learning environment that motivated them to accomplish their learning tasks and obtain the intended learning outcomes. Students also expressed that the GBL and gamification teaching methods’ content increased and sustained their attention and their interest in the learning approach. Game scenarios, graphics, game play, activity-oriented assignments, and competition may have captured their interest while learning, whilst increasing their motivation to learn more. These attributes in the game-based learning conditions may have produced a sense of connection between the learning content and the students [87] during the learning process. Interestingly, students expressed a sense of self assurance and increased self-esteem skills when using GBL and gamification teaching methods, helping them to avoid irresponsible sexual behaviour that could affect their current and future health outcomes.

#### 4.2.2. Attitude

Pedagogy of GBL may be effective in delivering the intended learning outcome because of the positive student attitudes toward the teaching methods and learning experiences [88]. Participants evaluating their attitudes toward behaviour change and instructional methods reported increasing positive attitudes and awareness of sexual health matter, claiming that the knowledge obtained helped to change current negative attitudes from engaging in irresponsible sexual behaviour to behaving in a sensible manner, which was in direct contrast to those in the control condition. The positive attitudes reported by the GBL and Gamification groups led to an increased interest and participation, as well as feelings of fun and enjoyment. This is consistent with recent studies showing that positive emotions and feelings are learned through GBL and gamification [21,89]. Behaviors learnt, copied, adopted, and/or observed by adolescents are practiced during the adolescence stage and can remain for a lifetime [90].

Our focus group discussions concur with the results presented by Naik [88], as participants in the traditional teaching method group reported that the method was not as effective. The learning was reportedly passive and unexciting. They understood that there are social-cultural issues related to sexual health matters in their community, but the traditional teaching approach disguised important information which resulted in negative attitudes towards the traditional teaching approach. The GBL and gamification groups, on the other hand, were able to be self-directed in learning the information provided. The findings concur with previous reports [91] and confirm that GBL and gamification are useful pedagogies for learning sexual health matters as they consider social and cultural sensitivity.

#### 4.2.3. Knowledge

The Knowledge construct from the MAKE framework confirmed the results from our ASHLT, which reported that GBL and gamification teaching methods are effective for the acquisition of sexual health knowledge. The participants evaluated the teaching methods as effective and felt that their knowledge was enhanced. Their counterparts were less positive about the traditional teaching method. According to Kapp [92], the “game” structure in the GBL and gamification methods is a motivating factor that fosters learning. Investigating specific game mechanics, and how this relates to motivating and engaging students in digital sex education games, may provide a deeper understanding of the learning outcomes. Our results coincide with previous findings proposing that gamification could be a promising approach for learning about healthy sexual behaviour [83].

The students perceived that GBL and gamification are pedagogies that are easy and friendly ways to enhance sexual health knowledge. According to the students, the sexual health knowledge acquired from the game-based learning methods was very important for their current and future life, which is supported by previous findings [31]. The knowledge gained is applicable to underserved community adolescents who have the highest likelihood of engaging in unhealthy sexual behaviour that can affect their health and contribution to social, economic, and political development [13,90]. During the focus group sessions, the students reported that the GBL learning applications effectively disseminated knowledge commensurate with digital generations and suggested that these methods be used to deliver sexual health knowledge to schools throughout the country, as they are effective and user-friendly.

#### 4.2.4. Engagement

Our findings showed high engagement for the students participating in the GBL and gamification conditions, whereas the students were disengaged and less immersed in traditional teaching classes, as reported in the focus group interviews. The interactivity of the game allows for active player participation during the teaching and learning process (viewing the game scenarios or stories, game play, attempting questions, and reading), and games offer an active learning environment in practice that can increase engagement [93]. The qualitative results obtained from the FGI reports were in accordance with our ASHLT assessment findings. The GBL and gamification methods were found to be engaging during learning and during the interviews, student replies were positive, explaining that the games gave them more tasks to perform, leading them to focus and concentrate on learning.

The research team also observed students enjoying the GBL and gamification teaching sessions and noted that they were reluctant to leave the computer lab when the training ended. Providing autonomy and opportunities for students to engage in challenges that are aligned with their skill levels fosters student engagement in the learning process [94]. These elements are fundamental components of the GBL and gamification methods and therefore it is not surprising that students were engaged. What is interesting is the link between engagement and learning in this study. Hew [56] supports our assumption that game mechanics engage learners who perform more difficult tasks, therefore fostering learning. In contrast, the students subjected to traditional teaching methods expressed the more negative feelings of being frustrated and bored and a lack of autonomy, resulting in shyness and a fear to ask questions in the class.

### 4.3. Limitations

This study is limited by the sample size and the homogeneity of the samples (one school in one city). This limits the generalizability of the results. Future explorations into gamification and game-based learning teaching methods should evaluate differences and benefits across larger and more diverse populations. Limited access to the computer lab, slow and unreliable internet connection speeds, and a lack of computer resources resulted in time constraints for playing the game. All of these issues were noted during the digital game-based learning sessions, though the students continued to be engaged, and our findings suggest that despite the limitations of technology, adolescents prefer the GBL and gamification teaching methods. Our findings have highlighted the need for increased funding and resources in game-based learning applications in developing countries to ensure that future research can determine the benefits of these innovative teaching methods.

### 4.4. Implications for Research and Practice

#### 4.4.1. For Students

Games for sexual health education enable school adolescents to gain knowledge and understanding of sexual health matters, promote and sustain risk-minimizing behaviour, make informed choices on sex and relationships, and discourage adolescents from engaging in sex before marriage.

- Adolescents should use the games wisely to obtain the intended knowledge and skills that help them to reduce risky sexual behaviour during and after adolescents.
- Efforts to include the end users (adolescents) in the research and development of sexual education games will enhance the games and provide a user-friendly platform for the target audience.

#### 4.4.2. For Teachers

Teachers in schools play an important role in improving sexual and reproductive health learning and health outcomes among school adolescents.

- Teachers responsible for teaching sexual and reproductive health among adolescents must be adequately acquainted with the subject.
- Teachers should be given support and encouraged to initiate the application of innovative approaches such as digital games in sexual health education.
- Input from teachers when developing, implementing, and investigating the use of sexual education games in the classroom is vital and should be encouraged.

#### 4.4.3. At School Level

School is a formal setting in which sexual health education can be offered to larger numbers of adolescents of different age groups and levels of study. Therefore, schools play a major role in preparing children and adolescents during their transition to adulthood. Although many schools provide basic sexual health information and education to adolescents, the teaching methods appear to be ineffective due to many factors, including culture, religion, and beliefs.

- Technology implementation that aligns with current operating systems and frequent upgrades to software and hardware are required.
- Adolescents should be provided with the appropriate sexual and reproductive health information and education to equip them with the knowledge and skills to make informed choices on both social and sexual aspects of life.
- The subject matter needs to be compatible with the school setting, socially acceptable norms, and cultural values, as freely talking about sexuality and sexual behaviour publicly is not acceptable, and the subject can be taboo in certain populations.

#### 4.4.4. For Parents

Parents and family members have critical roles in the physical, emotional, and sexual development of adolescents.

- Parents should be active participants and engage in the development and implementation of sexual health education games.
- Parents are a vital resource for determining both short- and long-term impacts of health games and should be included in efforts to understand the outcomes of these innovative teaching approaches.

#### 4.4.5. For Policy and Decision-Makers

Policy and decision-makers formulate policies and guidelines on the best ways to provide sexual and reproductive health services to adolescents. Learning through games can be a very effective method of reinforcing the cognitive development of adolescents, which affects their decision-making about sexual behaviour.

- Efforts to remodel the current curriculum and apply innovative teaching methods such as sexual health education GBL and Gamification are greatly needed.
- Policy- and decision-makers are advised to emphasize and support the use of games for improving sexual health education without interfering with the traditional values and beliefs of the study community.
- Create open discussions and provide support for research-based evidence to ensure best practice models to teach sexual health education are developed.

## 5. Conclusions

These findings support the use of GBL and gamification teaching methods to effectively deliver and improve sexual health knowledge of secondary school adolescents. The results were supported by consistently positive responses to all MAKE constructs on the GBL and gamification teaching methods. The support of various stakeholders was instrumental to the development of our study findings and further supports the use of the participatory approach to develop GBL and gamification teaching methods. The study confirms that if used in a positive way, games can be powerful educational tools in low resource settings and regions where discussions about sexual issues are taboo for adolescents vulnerable to high-risk sexual behaviour.

When developed in conjunction with various stakeholders, social-cultural norms can be taken into consideration and provide greater potential for teaching sexual health education The impact of participatory design models in sexual health education includes social contributions, particularly in imparting knowledge to adolescents in regions plagued by years of sexual health problems, including HIV/AIDS prevention. Our research provides one of the very first applications of GBL and gamification to the teaching of sexual health literacy to adolescents and is likely to be the first in Africa. This initiative could provide a model for other countries. Finally, the present study informs the initiatives and government plans in place to improve learning processes in secondary schools, and the application of technology in teaching and learning.

Our research documents information on interventions for the effective teaching of adolescent sexual health education based on the social, cultural, and economic background of developing countries, particularly Africa, where traditional teaching methods are commonly used. To the best of our knowledge, research into the use of GBL and gamification in teaching is non-existent, not only in the sexual health education study context, but also for other subjects. To further comprehend the extent to which games could potentially improve sexual health education among school adolescents, the implementation, evaluation, and efficacy of the three teaching conditions in diverse cultural communities is warranted. We have plans for sexual health information to become accessible through various devices, such as computers, mobile smart phones, and tablets—and to be available on Facebook and a website.

## Figures and Tables

**Table 1:** Randomized control trial for this study.

120 students from three classes were randomly selected to participated in the study	40 students randomly assigned to experiment condition	**Pre-test:** students given ASHLT before learning	**Experiment:** students attended sexual health education delivered using GBL	**Post-test:** students were given the same ASHLT after learning
	40 students randomly assigned to experiment condition	**Pre-test:** students given ASHLT before learning	**Experiment:** students attended sexual health education delivered using gamification	**Post-test:** students were given the same ASHLT after learning
	40 students randomly assigned to control condition	**Pre-test:** students given ASHLT before learning	**Control:** students attended sexual health education delivered using traditional teaching	**Post-test:** students were given the same ASHLT after learning

Notes: ASHLT = Adolescent Sexual Health Literacy Test.

**Table 2:** Descriptive statistics of the students and SES and media usage characteristics (*N* = 120).

**Gender**	**Frequency (%)**
Male	63 (52.5%)
Female	57 (47.5%)
**Age**	**Mean (SD)**
Male	14.2 (0.924)
Female	13.9 (0.963)
**Living condition**	**Frequency (%)**
With both parents	80 (66.7%)
With father only	7 (5.8%)
With mother only	21 (17.5%)
With guardian only	12 (10%)
**Economic condition**	**Frequency (%)**
We are among the well-off in the area	24 (20%)
We are not rich, but we manage to live well	62 (51.7%)
We are neither rich nor poor, but just about average	34 (28.3%)
We struggle with the strict minimum required to make ends meet	0 (0%)
**Access to computer at school or home**	**Frequency (%)**
Yes	82 (68.3%)
No	38 (31.7%)
**Access to smart devices at school or home**	**Frequency (%)**
Yes	66 (55%)
No	54 (45%)
**Play of computer or mobile phone games**	**Frequency (%)**
Yes	77 (64.2%)
No	43 (35.8%)

Note: SES: Social Economic Status.

**Table 3:** Descriptive statistics (with means of the experimental groups in bold) and pairwise comparison of teaching methods for the Motivation constructs.

**Motivation**	**Component**	**Measure**	**TT**	**GBL**	**GM**	**Sig. Kruskal Wallis**	**Pairwise Comparison**		***p* Value**
							**TM1**	**TM2**	
	Attention	Mean	2.55	**4.43**	**4.40**	0.001	TT	GM	0.001
		SD	1.16	0.37	0.40			GBL	0.001
	Relevance	Mean	2.76	**4.47**	**4.55**	0.001	TT	GBL	0.001
		SD	0.73	0.32	0.41			GM	0.001
	Confidence	Mean	3.65	**4.63**	**4.42**	0.001	TT	GM	0.004
		SD	1.12	0.32	0.52			GBL	0.001
	Satisfaction	Mean	3.67	**4.53**	**4.56**	0.001	TT	GBL	0.001
		SD	0.82	0.38	0.30			GM	0.001

Notes: Each item was rated on a five-point Likert scale ranging from 1 (Strongly Disagree) to 5 (Strongly Agree). TM: Teaching Method.

**Table 4:** Descriptive statistics (with means of the experimental groups in bold) and pairwise comparison of teaching methods for the Attitude constructs.

**Attitude**	**Component**	**Measure**	**TT**	**GBL**	**GM**	**Sig. Kruskal-Wallis**	**Pairwise Comparison**		***p* Value**
							**TM1**	**TM2**	
	Affective Attitude	Mean	3.64	**4.69**	**4.84**	0.001	TT	GBL	0.001
		SD	1.03	0.35	0.19			GM	0.001
	Cognitive Attitude	Mean	3.51	**4.76**	**4.77**	0.001	TT	GM	0.001
		SD	0.94	0.25	0.22			GBL	0.001

Notes: Each item was rated on a five-point Likert scale ranging from 1 (Strongly Disagree) to 5 (Strongly Agree). TM: Teaching Method.

**Table 5:** Descriptive statistics (with means of the experimental groups in bold) and pairwise comparison of teaching methods for the Knowledge constructs.

**Knowledge**	**Component**	**Measure**	**TT**	**GBL**	**GM**	**Sig. Kruskal-Wallis**	**Pairwise Comparison**		***p* Value**
							**TM1**	**TM2**	
	Importance of knowledge	Mean	3.13	**4.79**	**4.84**	0.001	TT	GBL	0.001
		SD	1.13	0.23	0.20			GM	0.001
	Effectiveness of knowledge	Mean	2.85	**4.76**	**4.88**	0.001	TT	GBL	0.001
		SD	0.95	0.34	0.26			GM	0.001
	Application of knowledge	Mean	3.97	**4.78**	**4.84**	0.001	TT	GBL	0.001
		SD	0.85	0.35	0.28			GM	0.001

Notes: Each item was rated on a five-point Likert scale ranging from 1 (Strongly Disagree) to 5 (Strongly Agree).

**Table 6:** Mean (SD) of teaching methods for the Engagement constructs.

**Engagement**	**Component**	**Measure**	**TT**	**GBL**	**GM**	**Sig. Kruskal-Wallis**	**Pairwise Comparison**		***p* Value**
							**TM1**	**TM2**	
	Emotional engagement	Mean	2.77	4.63	4.67	0.001	TT	GBL	0.001
		SD	0.96	0.33	0.28			GM	0.001
	Cognitive engagement	Mean	2.98	4.67	4.64	0.001	TT	GM	0.001
		SD	0.79	0.40	0.37			GBL	0.001

Notes: Each item was rated on a five-point Likert scale ranging from 1 (Strongly Disagree) to 5 (Strongly Agree).

## Appendix A. MAKE Evaluation

### Survey Questionnaire

**Measure**	**SD**	**D**	**N**	**A**	**SA**
**MOTIVATION: Attention Statement**					
There was something interesting at the beginning of the instructional method that got my attention.	☐	☐	☐	☐	☐
The teaching approach used is eye catching.	☐	☐	☐	☐	☐
The quality of activity in the teaching method holds my attention.	☐	☐	☐	☐	☐
The design of the teaching method looks appealing.	☐	☐	☐	☐	☐
**MOTIVATION: Relevance Statement**					
I could relate the content taught through this method to things I have thought about in my own future life.	☐	☐	☐	☐	☐
The content taught through this approach will be useful during my adolescent period.	☐	☐	☐	☐	☐
The content and instructional style convey the impression that the course is worth knowing.	☐	☐	☐	☐	☐
The content in the teaching approach will be useful to me.	☐	☐	☐	☐	☐
**MOTIVATION: Confidence Statement**					
I could really understand quite easily of the material taught through this teaching method.	☐	☐	☐	☐	☐
The exercises in this teaching approach were too easy.	☐	☐	☐	☐	☐
The good organization of the content helped me be confident that I would learn better in this approach.	☐	☐	☐	☐	☐
The teaching approach was simpler to understand than I would like for it to be.	☐	☐	☐	☐	☐
**MOTIVATION: Satisfaction Statement**					
I really enjoyed learning with this teaching method.	☐	☐	☐	☐	☐
It was a pleasure to learn sexual health behaviors through this pedagogy.	☐	☐	☐	☐	☐
Completing the exercise in this teaching method gave me a satisfying feeling of accomplishment.	☐	☐	☐	☐	☐
I learned somethings that were surprising or unexpected with this teaching method.	☐	☐	☐	☐	☐
**ATTITUDE: Affective Attitude Statement**					
The instructional approach increases my participation in the sexual health education.	☐	☐	☐	☐	☐
I feel happy to be taught sexual health education through this teaching method.	☐	☐	☐	☐	☐
The teaching method used makes sexual health education more interesting.	☐	☐	☐	☐	☐
The teaching method I attended is appropriate for the delivery of sexual health skills.	☐	☐	☐	☐	☐
The teaching method used is an ideal for sexual health education.	☐	☐	☐	☐	☐
**ATTITUDE: Cognitive Attitude Statement**					
The teaching method I attended enhanced my understanding of sexual health behaviour issues.	☐	☐	☐	☐	☐
The instructional approach explained the sexual health learning materials very well.	☐	☐	☐	☐	☐
I found the instructional worthwhile in the sexual health course.	☐	☐	☐	☐	☐
The method of instruction used in the sexual health course I attended was interesting.	☐	☐	☐	☐	☐
The teaching method used arouses interest in sexual health education program.	☐	☐	☐	☐	☐
**KNOWLEDGE: Importance of Knowledge Statement**					
The teaching approach enhanced my knowledge about STDs, STIs, and HIV/AIDS.	☐	☐	☐	☐	☐
I gained knowledge about reproductive health through this teaching method.	☐	☐	☐	☐	☐
I gained knowledge about sexual decision-making through the method of instruction.	☐	☐	☐	☐	☐
The instructional method used helped me gain knowledge about good manners and personal hygiene.	☐	☐	☐	☐	☐
**KNOWLEDGE: Application of Knowledge Statement**					
I will apply the sexual coercion and assault knowledge taught through this approach.	☐	☐	☐	☐	☐
The topic of responsible sexual behavioral practices taught in this approach seems very relevant.					
The method of teaching helped me gain knowledge on dealing with peer pressure during adolescence.	☐	☐	☐	☐	☐
**KNOWLEDGE: Effectiveness of Knowledge Statement**					
The method of instruction used is very active as it helped my understanding of the importance of abstinence.	☐	☐	☐	☐	☐
The teaching approach is very effective as it extended my existing understanding of the course.	☐	☐	☐	☐	☐
This teaching method is very effective in imparting knowledge in sexual health education programs.	☐	☐	☐	☐	☐
**ENGAGEMENT: Emotional Engagement Statement**					
The teaching method I attended it was very easy to understand the learning contents.	☐	☐	☐	☐	☐
I have been effective in this course as the method of instruction was engaging.	☐	☐	☐	☐	☐
The teaching method used facilitates my active participation in the subject taught.	☐	☐	☐	☐	☐
The method of instruction used caught my attention during the course.	☐	☐	☐	☐	☐
This method allowed my expression of thoughtful ideas relevant to the course.	☐	☐	☐	☐	☐
The instructional approach used during the course made me interested.	☐	☐	☐	☐	☐
**ENGAGEMENT: Cognitive Engagement Statement**					
I demonstrated my interest and enthusiasm as well as use of positive humor during the course.	☐	☐	☐	☐	☐
This teaching method is relevant for engaging students in the sexual education course.	☐	☐	☐	☐	☐
The teaching strategy used enhanced my engagement in the course.	☐	☐	☐	☐	☐
I focused on the learning activity given in this teaching approach.	☐	☐	☐	☐	☐
Notes: SD = Strongly Disagree (1 point); D = Disagree (2 points); N = Neutral (3 points); A = Agree (4 points); and SA = Strongly Agree (5 points).					

This survey instrument was adapted from various prior studies [71,72,73] to reflect the research objectives.

## Appendix B. MAKE Evaluation

### Focus Group Interview Guide

The main research question was how and why think the teaching methods used in sexual health education has Motivated to learn, promoted change of Attitude, enhanced Knowledge gain, and Engaged during learning. Specifically, the FGI protocol asked:

- Do you think the teaching method motivated you to learn? How and why?
- Do you think the teaching method affect your learning attitude? How and why?
- Do you think the teaching method enhanced you to gain the intended knowledge? How and why?
- Do you think the teaching method engaged you during learning? How and why?
